# Use of molecular biology tools for rapid identification and characterization of *Pasteurella* spp

**DOI:** 10.14202/vetworld.2018.1006-1014

**Published:** 2018-07-29

**Authors:** Ashraf M. Abbas, Dalia A. M. Abd El-Moaty, Eman S. A. Zaki, Elham F. El-Sergany, Nadine A. El-Sebay, Hala A. Fadl, A. A. Samy

**Affiliations:** 1 Genetic Engineering Research Department, Veterinary Serum and Vaccine Research Institute, Cairo Egypt; 2 Aerobic Bacterial Vaccine Research Department, Veterinary Serum and Vaccine Research Institute, Cairo Egypt; 3 Anaerobic Bacterial Vaccine Research Department, Veterinary Serum and Vaccine Research Institute, Cairo Egypt; 4 Department of Microbiology and Immunology, Veterinary Division, National Research Center, Dokki, Egypt

**Keywords:** multiplex polymerase chain reaction, outer membrane protein H, *Pasteurella multocida*, restriction endonucleases analysis

## Abstract

**Aim:**

This study aimed to create rapid characterization and genotyping of *Pasteurella multocida* (PM) protocol using modern molecular biology techniques.

**Materials and Methods:**

Thirty bacterial isolates were characterized by capsular and somatic identification using conventional procedure followed by multiplex polymerase chain reaction (PCR), restriction endonucleases analysis (REA), and finally confirmed by sequence analysis. Two local vaccine strains and two field isolates were identified as PM Type A and B.

**Results:**

A total of 30 isolates were found positive for PM either morphologically and biochemically; however, multiplex PCR technique identified only 22 isolates as *Pasteurella* species using universal primers while 8 isolates were found negative for PM. 12 of 22 isolates (54%) were characterized at the same reaction into PM Type A, five isolates (23%) were Type B and the rest five isolates (23%) of tested isolates were negative for Types A, B, and D. Hemorrhagic septicemia Type B: 2 or B: 5 could be identified somatically within PM capsular serogroup B using PCR technique. Somatic characterization of PM was done using REA that could identify all PM Type A into A:1 and all PM Type B into B: 2. These protocols were verified for its accuracy and reliability by sequence analysis of two vaccine strains of PM Type A and B that were characterized previously by biochemical and serological methods as well as two selected isolates from the 22 positive isolates representing PM Type A and B.

**Conclusion:**

PCR and REA could confirm the identity of PM and provide a rapid and reliable characterization in comparison with biochemical analysis and conventional serotyping that may take up to 2 weeks. Hence, they can reduce the time needed for polyvalent vaccine production and when the reference antisera are unavailable. Moreover, the identity of Omp-H for vaccine and field strains may provide better data to control Pasteurellosis in Egypt.

## Introduction

Members of the family Pasteurellaceae are associated with some diseases; many of them show respiratory signs [1]. A member of this family - *Pasteurella multocida* (PM) - is the causative agent of several diseases that have a great economic impact on animal and poultry production. Different *Pasteurella* species still represent severe threats in Egypt. They are associated with the potential cause of pneumonia in domestic sheep and goats and shipping fever complex, hemorrhagic septicemia (HS) in cattle, fowl cholera (FC) in domesticated, and wild birds [2,3]. Serotyping of PM isolates depends on the typing systems; capsular typing was done by Carter’s IHA system [4] and the somatic typing using 1 h boiled supernatant agar gel precipitation [5]. As PM is a heterogeneous species, typing of Carter’s HIA system depends on differentiation by serology into five types of capsular serotype (A, B, D, E, and F) [4]. The phenotypic characterization systems using morphology, biochemical and serotyping are very much laborious and time-consuming [6]. Even after capsular and somatic antigen determination, some of the isolates cannot be differentiated because they may react similarly in both the antigens [7]. Furthermore, the agglutination of homologous antiserum may fail. The agglutination failure of serogroups A, D, and F with homologous antisera is one of the main causes of reduced sensitivity in this phenotypic test [8]. Passive hemagglutination has a substantial concern as that test can be rendered ineffective by the loss of PM capsule after repeated subcultures *in vitro* [9]. Several molecular techniques have been developed for PM typing such as restriction endonuclease analysis (REA), ribotyping, pulsed-field gel electrophoresis, and polymerase chain reaction (PCR) based fingerprinting [10,11]. The PCR-based techniques have provided the alternative methods of characterization to overcome the limitations of phenotyping [7]. Primers for PM were designed to detect a fragment of the *KMT1* gene encoding the outer membrane protein producing an amplification product unique to all strains of PM [12,13]. The comparative analysis of the genetic organization of region 2 of the capsule biosynthetic loci of the five PM capsular serogroup (serogroups A, B, D, E, and F) showed that sequences within *hyaD*, *bcbD*, *dcbF*, *ecbJ*, and *fcbD* have been determined as highly specific for their respective serogroup. Notably, this PCR-based system was not affected by the geographical distribution of isolates that may also help to clarify the distinction between strains from closely related serogroups A and F [14]. The gene *hyaD*– *hyaC* was an ideal amplification target for PCR because hyaluronic acid is a principal component of Type A capsule [15]. HS associated PM strains belonging to capsular serogroup B form a very closely related group but are distinguishable using whole genome analysis. Identification of 96 genes unique to the HS associated strains that need a characterization of these genes may elucidate their role in disease pathogenesis, virulence and host specificity [16]. A primer pair designed from the sequence of the clone 6b (KTT72 and KTSP1) could specifically amplify a DNA fragment from types B:2, B:5, and B:2, 5 [12]. Outer membrane protein H (OmpH), the major Omp has a role in the immunogenicity and pathogenicity of PM isolates [17-19]. PCR-RFLP based on *ompH* gene was potentially a useful method for typing of PM for studying the epidemiology of PM infections in avian isolates [20]. Further typing of PM isolates with REA of *OmpH*-PCR product using restriction enzymes *Dra I and Hinf I* could generate distinct profiles for three serotypes A:1, A:3, and B:2 [21].

The unavailability of antisera used for capsular typing and the urgent need for characterization of field isolates of recent infection or before vaccine preparation is the main obstacle. Furthermore, it is important for identifying and genotyping of field isolates in the early stages of infection or before the production of efficient polyvalent *Pasteurella* vaccines that need important justifications.

The aim of this study was to overcome the difficulty in obtaining antisera by rapid and alternative methods for detection and characterization of *Pasteurella* different types by application of molecular biology tools.

## Materials and Methods

### Ethical approval

The approval from the Institutional Animal Ethics Committee to carry out this study was not required as no invasive technique was used.

### Bacterial isolates

Thirty bacterial isolates were isolated from suspected *Pasteurella* outbreaks of different hosts (sheep, cattle, buffalo, and poultry) from different governorates during the past 10 years in Egypt. These isolates were identified by the department of aerobic bacterial vaccines in VSVRI and kept lyophilized till used in this study. Ampoules were opened under the sterile condition and contents reconstituted in brain heart infusion, which afterward was inoculated on blood agar media with 5–10% defibrinated sheep blood, and MacConkey agar media, and incubated aerobically at 37°C for 18–24 h.

A total of 30 isolates were identified by morphological and biochemical methods [6,22]. One field isolate designated (PM/VSVRI/99) was collected from sheep pneumonic lungs from Sakha farm, Kafer El-Sheikh in 1999 while another isolate designated (PM/VSVRI/2015) was collected from buffalo lungs, Cairo abattoir in 2015. Brucella vaccine strain was gratefully obtained from Antigens and Sera Department, VSVRI, Cairo, Egypt to be used as negative control.

Two vaccine strains were used as positive control in this study. Strain (PM/VSVRI/1962) used in Pneumobac^®^ vaccine (VSVRI, Egypt) was isolated from cattle during HS outbreak in Egypt since 1962 and identified as PM Type B [4,5,23,24]. Strain (PM/VSVRI/2004) used in FC vaccine^®^ was collected from poultry heart blood, Sharkia, Egypt during 2004 outbreak of v. It was characterized and identified as PM Type A [4,22].

### DNA extraction

DNA from all isolates was extracted using Gene Jet Genomic DNA purification Kit (#k0721), Thermo Scientific, USA according to the manufacturer’s protocol.

### Primers sets

Various sets of published primers (Tables-1,2 and 3) were used for molecular characterization of PM.

**Table-1:** Sequences of the oligonucleotides used in the *P. multocida* multiplex capsular PCR typing assay [14].

Serogroup	Primer description	Primer sequence	Annealing temperature	Amplimer size (bp)
*P. multocida* universal primers	KMT1 T7 -F	5’- ATC-CGC-TAT-TTA-CCC-AGT-GG-3’	55°C	460
	KMT1SP6-R	5’- GCT-GTA-AAC-GAA-CTC-GCC-AC-3’		
A	CAPA-F	5’- TGC-CAA-AAT-CGC-AGT-CAG-3’	55°C	1,044
	CAPA-R	5’- TTG-CCA-TCA-TTG-TCA-GTG-3’		
B	CAPB-F	5’- CAT-TTA-TCC-AAG-CTC-CAC-C-3’	55°C	760
	CAPB-R	5’- GCC-CGA-GAG-TTT-CAA-TCC-3’		
D	CAPA-F	5’ – TTA-CAA-AAG-AAA-GAC-TAG-GAG-CCC-’3	55°C	657
	CAPA-R	5’CAT-CTA-CCC-ACT-CAA-CCA-TAT-CAG-’3		

*P. multocida=Pasteurella multocida*, PCR=Polymerase chain reaction

**Table-2:** Oligonucleotides sequences used in hemorrhagic septicemia causing type-B-specific PCR assay [12].

Serogroup	Primer description	Primer sequence	Annealing temperature	Amplimer size (bp)
B2 or B5	KTT 72- F	5’- AGG-CTC-GTT-TGG-ATT-ATG-AAG 3’	55°C	620
	KTSP 72- R	5’- ATC-CGC-TAA-CAC-ACT-CTC-3’		

PCR=Polymerase chain reaction

**Table 3:** Sequences of the oligonucleotides used in amplification of *ompH* gene of *P. multocida* [25].

Serogroup	Primer description	Primer sequence	Annealing temperature	Amplimer size (bp)
*P. multocida*	OmpHF	5’- GCG-TTT-CAT-TCA-AAG-CAT-CTC -3’	56°C	1000
*ompH* gene	OmpHR	5’- ATG-ACC-GCG-TAA-CGA-CTT-TC -3’		

P. multocida=Pasteurella multocida

### Multiplex PCR amplification for universal detection and capsular serogrouping of PM

Multiplex PCR reaction was performed as following protocol: (25 µL 2× Dream Taq Green PCR Master Mix (Fermentas), 100 pmol of each forward and reverse primers of the four sets mentioned in Table-1 [14], 2 µL template DNA, and nuclease-free water up to 50 µL. The amplification reactions were performed using thermal cycler Perkin Elmer Gene Amp. PCR system 9700. The thermal cycler was adjusted to initial denaturation at 95°C for 5 min, then 30 cycles at 95°C for 1 min, 55°C for 1 min, and 72°C for 1 min followed by final extension at 72°C for 7 min. The amplified products were analyzed by electrophoresis using 1% agarose gel and visualized by ultraviolet transilluminator after staining the gel with ethidium bromide stain (Fisher). The product size was measured using Gene Ruler 100 bp Plus DNA Ladder (Thermo Scientific). HS-causing Type-B-specific PM PCR assay was performed using the same thermal and cyclic conditions multiplex PCR described above but with using one primer set mentioned in Table-2 [12].

### Amplification of ompH gene [21]

A 50 µL reaction mixture was prepared using 100 pmol of each forward and reverse primers mentioned in Table-3 [25]. The amplification reaction was carried out with an initial denaturation at 94°C for 5 min, followed by 35 cycles at 94°C for 15 s, 56°C for 1 min, and 72°C for 1 min, the final extension at 72°C for 10 min for all 22 PM positive isolates. The agarose gel extraction kit Qia-quick (Qiagen, Germany) was used for purification of *ompH* gene PCR products (1000 bp) to be used for REA and sequence analysis.

### REA of amplified ompH gene PCR product (serotypes A:1, A:3, and B:2)

The purified PCR products of *ompH* gene of all PM standard vaccine strains and isolates were digested by restriction enzymes Fast digest *HinfI and DraI*, (Thermo, USA) according to the manufacturer’s instructions. The reactions were analyzed by electrophoresis 1.5% agarose stained with ethidium bromide.

### Sequence analysis

Two isolates representing PM Types A and B depending on multiplex PCR results, as well as the standard vaccine strains (PM/VSVRI/1962 and PM/VSVRI/2004) used in this study, were selected for sequence analysis using the same *ompH* gene PCR primers (Macrogen Inc. Seoul, Korea). These isolates represented different geographical locations, different collection time, and different hosts.

The resulted *ompH* gene sequences of four selected isolates were aligned with *ompH* gene sequence on both nucleotides and deduced amino acid sequences data available in GenBank of serotypes A, B, and D of PMs. Serotype A includes X-73 (U50907), A:1(AY606823), (HM582887), PAC-21-13/08 (HM348767), MCK-13 (KP212395), P-1059 (EF203903), PAB-15-6/08 (HM348766), A2 (U52200), A4 (U52201), A5 (U52202), A6 (U52203), A7(U52204), A8 (U52205), A9 (U52206), A10 (U52207), A11 (U52208), A13 (U52209), A13 (2) (U52210), A14 (U52211), A15 (U52212), and Cu (U52213). Serotype B includes P52 (EU016232), HB01 (CP006976), Omsk-13 (KP212396), OH1905 (CP004392), and HN06 (CP003313), and serotype D (AY864815). Multiple alignments were done using Bioedit program, MEGA5 program for phylogenetic analysis and MegAlign program available in DNASTAR Lasergene software package for similarity percentage.

## Results

All the 30 isolates were characterized and identified as PM by morphological and biochemical characteristics. All the isolates were found as small Gram-negative rods and coccobacilli, oxidase, catalase, and ornithine decarboxylase positive, urease negative and fermented glucose, mannitol, and sucrose but not lactose and showed no visible growth on MacConkey agar and reduced nitrate to nitrite.

### Multiplex capsular PCR typing system

**Figure-1:**
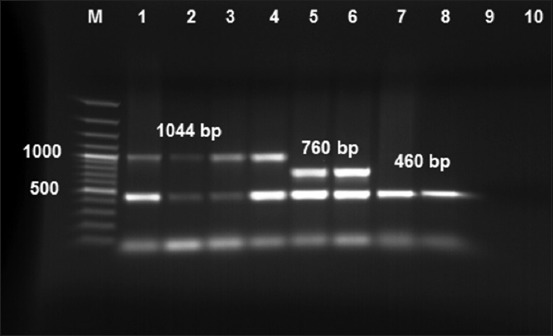
*Pasteurella multocida* multiplex capsular polymerase chain reaction (PCR) typing system. All tested isolates lanes (1–8) shared the same band at 460 bp of *KMT1* gene general for all *P. multocida* serotypes. M: 100 bp DNA ladder. Lane (1): PM/VSVRI/2004 vaccine strain Type A. Lane (2): PM/VSVRI/2015. Lane (3–4): Field isolates showing specific band for Type A at 1044 bp of *hyaD-hyaC* gene and Lane (5): PM/VSVRI/1962 vaccine strain Type B Lane (6): PM/VSVRI/99 showing specific band for Type B at 760 bp of *bcbD* gene. Lane (7-8): Isolates showing only 460 bp general for PM but negative for Types A, B, and D. Lane (9): Brucella negative control and Lane (10): PCR negative control.

A total of 30 isolates were tested by multiplex PCR with the two vaccine strains (PM/VSVRI/1962 and PM/VSVRI/2004) as positive controls. 22 isolates of PM isolates as well as the positive controls showed the expected PCR products (460bp) with PM specific primers (KMT1 primers) (Figure-1), while eight isolates showed negative PCR results. 12 isolates of the 22 positive PM isolates showed specific band 1044 bp for *Pasteurella* Type A (Lane 2–4) as well as PM/VSVRI/2004 Type A vaccine strain (Lane 1). Five isolates of the 22 positive isolates showed specific band for Type B at 760 bp (Lane 6) as well as PM/VSVRI/1962 Type B vaccine (Lane 5). The remaining five isolates of 22 positive isolates were negative for Types A, B, and D and showed only the general band of PM at 460 bp (Lane 7–8). The negative bacterial control of *Brucella* and the negative PCR control showed negative PCR results (Lane 9, 10).

### HS-causing type-B-specific PCR assay

**Figure-2:**
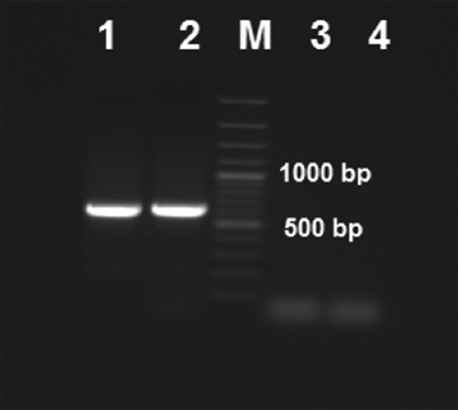
Hemorrhagic septicemia - causing Type-B-specific polymerase chain reaction assay for identifying Type B cultures either Type 2 or 5 of *Pasteurella multocida* by amplification of 620 bp fragment. M: 100 bp DNA ladder. Lane (1): PM/VSVRI/1962 vaccine strain Type B and Lane (2): Field isolate PM/VSVRI/99 showed 620 bp specific bands. Lane (3): PM/VSVRI/2004 vaccine strain Type A and Lane (4): *Brucella* negative control showed no amplification.

The five field isolates and vaccine strain Type B showed that the specific band for Type B (760 bp) was identified for HS-causing Type B serotypes of isolated PM (Figure-2). The cultures of Type B with dominant somatic antigen being either Type 2 or 5 were identified by the amplification of a 620 bp fragment with the KTSP61 and KTT72 primers. The vaccine strain PM/VSVRI/1962 with all five PM Type B isolates showed positive 620 bp fragment specific for Type B either 2 or 5 (Figure-2).

### PCR Amplification of ompH gene

**Figure-3:**
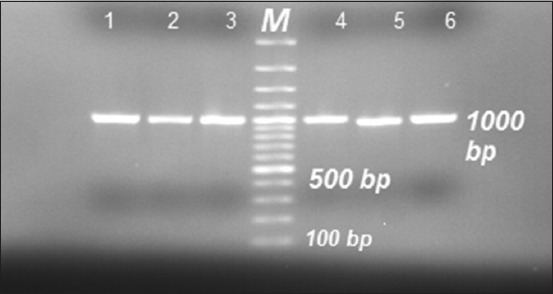
Polymerase chain reaction products of *ompH* gene approximately 1000 bp for all vaccine strains and field isolates. M: 100 bp DNA ladder. Lane (1): PM/VSVRI/2004 vaccine strain Type A, Lane (5): PM/VSVRI/1962 vaccine strain Type B, Lane (2–4): Three field isolates PM Type A including PM/VSVRI/2015, and Lane (6): PM/VSVRI/99 Type B isolate.

*ompH* gene of 12 PM Type A isolates, as well as 5 PM Type B with the two vaccine strains, were amplified by PCR using OmpHF and OmpHR primers producing PCR amplicon of approximately 1000 bp (Figure-3).

### REA

**Figure-4:**
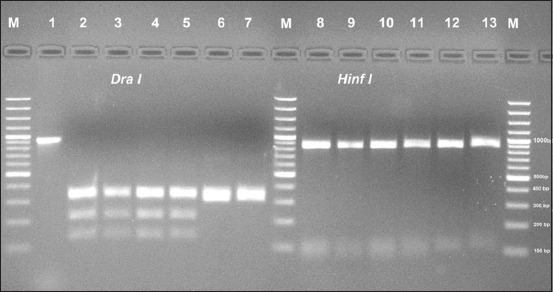
Restriction fragment length polymorphism of *ompH* - polymerase chain reaction (PCR) products of 1000 bp. Lanes (2–7) showed *ompH* fragment digested with *Dra I*. Lanes (8–13) showed *ompH* fragments digested with Hinf I restriction enzymes. M: 100 bp DNA ladder. Lane (1): Undigested *ompH*-PCR product. Lane (2): PM/VSVRI/2004 vaccine strain Type A, Lanes (3–5): Field isolates Type A including PM/VSVRI/2015, Lanes (6): PM/ VSVRI/1962 vaccine strain Type B isolates, Lane (7): PM/VSVRI/99 Type B isolate *Dra I* digests. Lane (8): PM/VSVRI/2004 vaccine strain Type A, Lanes (9–11): Field isolates Type A including PM/VSVRI/2015, Lane (12): PM/ VSVRI/1962 vaccine strain Type B, and Lane (13): PM/ VSVRI/99 Type B isolate *Hinf I* digests.

REA was performed for all *Pasteurella* Type A and B isolates as well as the two vaccine strains using *Dra I* and *Hinf I* restriction enzymes. All tested *Pasteurella* Type A showed the same pattern and fragment length (340, 320, 210, and 130 bp) with *Dra I* restriction enzyme, while they showed 3 fragments by *Hinf I* restriction enzyme with lengths (70, 80, and 850 bp) (Figure-4).

*Pasteurella* Type B isolates showed different profile when using *Dra I* enzyme to give three overlapping fragments at lengths (370, 320, and 300 bp) and two fragments (920 and 80 bp) when *Hinf I* enzyme was used (Figure-4).

### Sequence and phylogenetic analysis

The amplified *ompH* gene (1000 bp) fragments were purified for two Type A (PM/VSVRI/2004 vaccine strain and PM/VSVRI/2015 field isolate) and 2 PM Type B strains (PM/VSVRI/1962 vaccine strain and PM/VSVRI/99 field isolate) and sent for sequence analysis. The resulted sequences were submitted to GenBank with accession no (KY403514, KY403515, KY436382, and KY403516), respectively.

The computational restriction map of obtained sequences of PM/VSVRI/2004 vaccine strain and PM/VSVRI/2015 of Type A:1 sequences revealed the presence of three restriction sites specific for *Dra I* yielding 4 fragments with lengths (338, 321, 210, and 131 bp). Furthermore, the restriction map showed the presence of 2 restriction sites specific for *Hinf I* that yields three fragments with lengths (79, 72, and 850 bp). Restriction map of Type B2 sequence showed the presence of two restriction sites for *Dra I* giving fragment length (321, 305, and 374) while for *Hinf I* it showed unique site yielding two fragments (920 and 80).

The homology percentage between PM/VSVRI/2004vaccine strain and PM/VSVRI/2015 field isolate was 100% with A:1 and 98.6–98.3% with X73 strain on nucleotide and amino acid level, respectively. Furthermore, the homology percentage between PM/VSVRI/1962 vaccine strain and PM/VSVRI/99 field isolate was 100% with P52 (serotype B: 2) strain. The identity between Egyptian Types A and B strains was 78.6% on the amino acid level while it was 83.9–92% with Type D, respectively.

**Figure-5:**
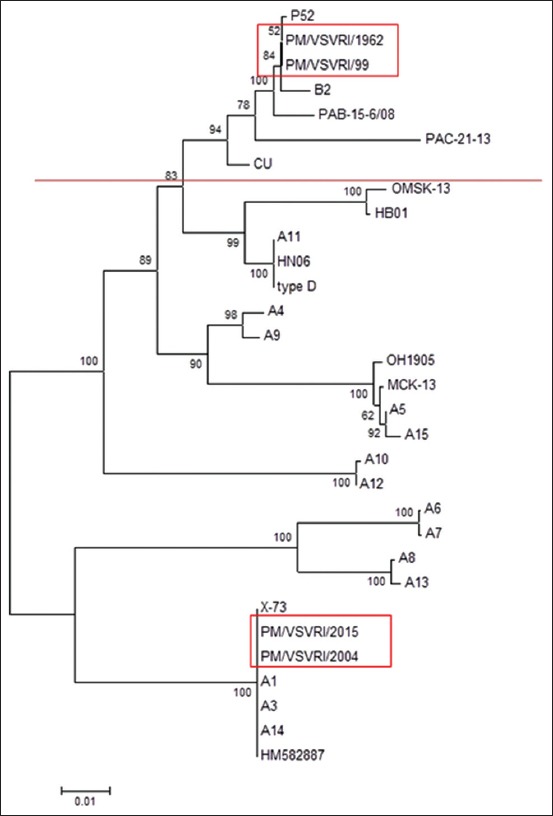
Rooted phylogenetic tree of nucleotide sequences of *Pasteurella multocida ompH* gene of different serotypes.

The phylogenetic dendrograms of both nucleotide sequence and deduced amino acid showed that PM/VSVRI/2004 and PM/VSVRI/2015 were clustered with A1, X73, A3, A14, HM582887 PM serotype A, and also confirmed that PM/VSVRI/99 and PM/VSVRI/1962 were clustered with P52 (serotype B:2) (Figure-5).

**Figure-6:**
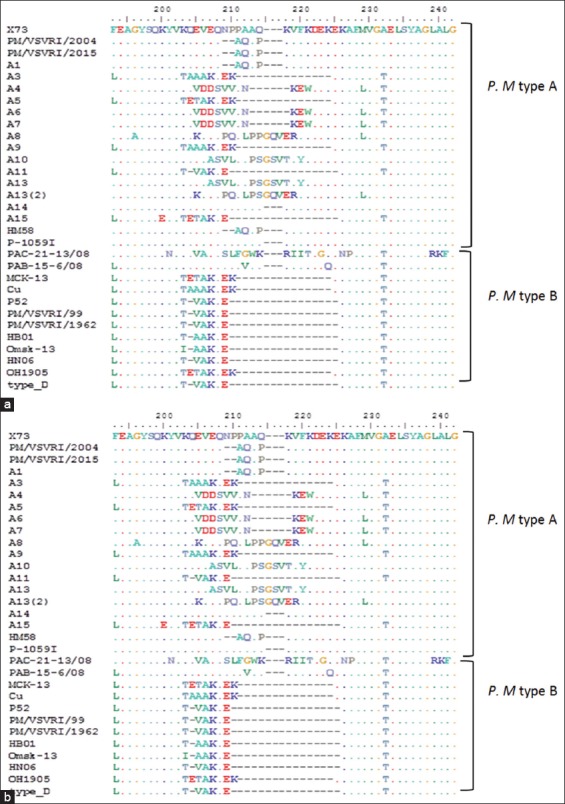
(a and b) Amino acid multiple alignments of OmpH of *Pasteurella multocida* of Egyptian strains with different serotypes of *P. multocida*.

The multiple alignments of deduced amino acid sequences of our two Type A Egyptian strains (PM/VSVRI/2004 and PM/VSVRI/2015) and two Type B Egyptian strains (PM/VSVRI/99 and PM/VSVRI/1962) with 20 Type A strains, 5 Type B, and 1 Type D strain revealed the presence of two major areas of variation (Figure-6). The first distinct region was 62–91 a.a residues -numbering is according to X-73 amino acid sequence - while the second area was 197–220 a.a residues. These areas represent two external loops L2 and L5, respectively.

Egyptian strains (PM/VSVRI/2004 and PM/VSVRI/2015) were identical to A:1, X-73, and HM58 and P-1059 strains at L2 loop. Other strains as A3 and A9 showed insertions at loop L2 and many deletions were detected between all serotype A subtypes as well as with serotype B strains. Strains (PM/VSVRI/99 and PM/VSVRI/1962) were identical to strain P52 at L2 region but different from other serotype B strains. The external loop L5 was an area of high variations and showed both deletions and insertions. The Egyptian strains (PM/VSVRI/2004 and PM/VSVRI/2015) and A:1 were identical but have two deletions at 206NP207 from X-73 strain.

Many deletions at L5 were detected between another serotype A subtypes, while three insertions were found at A8, A10, A13, and A13 (2). Furthermore, serotype B strains showed a high number of deletions with serotype A strains but with slight differences between serotype B different strains.

## Discussion

*Pasteurellosis* has long been recognized as a disease of major economic importance among live stocks and birds. *Pasteurellosis* confirmation seems to be difficult due to multiple clinical symptoms and time-consuming laboratory procedures. Furthermore, it is difficult to obtain a pure culture of PM from clinical isolates because of contaminants and/or death of organisms [26]. It has been observed that the detection of PM in the clinical material is greatly accelerated by the use of PCR technique and could yield isolation of the organism.

The advantages of the PCR compared with existing tests include better speed, sensitivity, specificity, and simplicity. It does not require culture or laboratory animals and is, therefore, safer as a result of the avoidance of handling live bacteria. The present study was aimed to establish a PCR assay specific for identification and typing of PM serotypes to be used for vaccine production. The present PCR method was designed to identify any PM strain by amplification of *KMT1* gene with genotyping of it depends on the cap loci at *hyaD*, *bcbD*, and *dcbF* that were highly specific for serogroups A, B, and D, respectively, gene of PM in a single step of multiplex PCR [12].

Although all the 30 field isolates were identified as PM either morphologically or biochemically, only 21 isolates had shown the specific 460 bp of *KMT1* gene amplification of PM. Eight isolates were negative for *KMT1* gene and all genes specific for Types A, B, and D tested in this study. The multiplex PCR could identify whether it is PM or not with its type at one reaction. These PCR results were supported with previously reported findings [14,27].

The majority of the PCR positive isolates were Type A (54%) while 23% were identified as Type B. Only five isolates showed only 460 bp of PM but negative for Types A, B, and D (Figure-1). Previous reports mentioned the existence of untypable PM strains [12], but we could not confirm the type of these 5 isolates due to the absence of Types F and E primers, but we suggest that these five isolates may be related to untypable PM strains as Types E and F were not recorded in Egypt up till now. All five PM Type B isolates showed that the specific band (760 bp) was confirmed to be HS-causing PM Type B either 2 or 5 serotypes showing positive 620 bp fragment using KTSP61 and KTT72 primers as mentioned previously [12].

REA of *ompH* gene (1 kb amplicon) was used to distinguish between PM serotype A:1, A:3, and B:2. Our results revealed that all the 12 Type A isolates were related to A:1 and while the five Type B PM isolates were related to B:2. Hence, *Dra I* and *Hinf I* restriction enzymes could be used for further characterization of both PM Types A and B local isolates [17,21].

Sequence analysis of *ompH* gene for two Egyptian isolates and two vaccine strains representing PM Types A and B were done for detailed characterization of PM isolates.

Many types of research have focused on 16S rRNA gene, but sequence analysis of this slowly evolving 16S rRNA gene is incapable of detecting the finer variation that exists between isolates from the same host species and which has previously been demonstrated by analysis of OMP profiles [28]. The presence of very different OMP profiles in isolates of the same or similar 16S types but from different host species indicated that cell-surface proteins are evolving more rapidly than the 16S rRNA gene and suggested a correlation between OMP diversification and host adaptation [29].

OMPs have been reported to be involved in virulence of the organism as colonization and invasion so; they are considered potential antigenic candidates [30]. OMPs of PM have been shown to stage pivotal role in host-pathogen interaction and disease processes based on functional characteristics. Then, identification of OMPs is critical to determine the protective antigens and to develop novel diagnostics after adequate evaluation of the host immunogenic reactivity as well as their antigenic potential [31].

The phylogenetic analysis of nucleotide sequence analysis clustered the two Egyptian Type A with A:1, X-73, A3, and A14 while the two Type B Egyptian strains were clustered with P52 and other B strains. The phylogenetic analysis has confirmed of results of REA. Furthermore, the computational restriction map of amplified OMP-H sequence analysis of PM/VSVRI/2004 and PM/VSVRI/2015 of serotype A:1 have confirmed the obtained results of REA as well as the restriction map of B:2 (PM/VSVRI/99 and PM/VSVRI/1962) nucleotide sequence showed the same profile. Hence, the sequence data have confirmed and validated all the previous results of multiplex PCR, HS-causing Type B PCR, and REA and supporting their reliability.

Interestingly, serotype A:1 strain PM/VSVRI/2004 was isolated from poultry at Sharkia governorate at 2004 while PM/VSVRI/2015 was isolated from buffalo at Cairo during 2015. The same situation was recorded between serotype B: 2, as PM/VSVRI/1962 was isolated from cattle early in 1962, while PM/VSVRI/99 was isolated from sheep at Sakha/Kafer El-Sheikh during 1999. Even though the A1 strain (KY606823) showed that 100% identity with the two Egyptian strains type A was isolated from duck in India. Consequently, the alignment between the whole genomes available on the GenBank database implied that the capsular type might affect the phylogenetic grouping rather than host species [32].

These findings may lead us to a conclusion that PM *Omp-H* gene from each serotype is stable in Egypt and it seems to be not affected either geographically, temporally or even the host which may be a promising indicator for controlling PM in the Egyptian field. The multiple alignments of deduced amino acid of our Egyptian strains with other universal Type A, B, and D serotypes revealed the presence of two major areas of variation. The first distinct region was 62–91 a.a residues, while the second area was 197–220 a.a residues and these areas represent two external loops L2 and L5, respectively. These findings were in unison with previous studies by other researchers [33,34].

Our results confirmed that L2 and L5 external loops are the regions of high variation where deletions and insertions or even substitutions occur. This may provide a reasonable explanation for the different antigenic and protective characteristics between different OmpH types (Figure-6). The differences were tended to be more noticeable at L5 than L2 in general, but L2 was identical between all Egyptian isolates and their reference strains. It was also noticed that within serotype A:1 strains there may be many differences as mentioned in the previous study [33] due to amino acid insertions at 205 LQP 206 in loop L5 at A1 with other insertions detected at A3, A4, and A6 that were not detected at our Egyptian A1 isolates. Hence, more molecular surveys are needed to check the appearance of different subtypes within the circulating serotypes in the Egyptian field.

When loop 2 and 5 amino acid sequences of X-73 OmpH were chosen to design synthetic peptides as vaccine, low titer of antibodies was induced by cyclic-L2 against itself and native protein in the whole cell lysate, but it was significantly immunogenic and protective without conjugation to a carrier protein, suggesting that both protective B-cell epitopes and T-cell epitopes were located in cyclic-L2. Meanwhile, the cyclic-L5 could not induce protection indicating that loop 5 does not contain protective epitopes. Furthermore, the study added that cyclic-L2 induced functional and conformationally relevant antibodies able to react with protective epitopes of OmpH at the cell surface of X-73 [17]. Therefore, the variations detected in L5 of all Egyptian isolates may be not significant as it does not contain protective epitopes. Another study found that some areas of the genomes had a higher concentration of single-nucleotide polymorphisms (SNPs) than others that could be due to interaction with the host immune system [35].

## Conclusion

Capsular multiplex PCR–REA assay was found to be very convenient and reliable technique for serogrouping of PM isolates within 6–8 h, in contrast to conventional serogrouping of the organism which is very time-consuming and requires the production and maintenance of a battery of hyperimmune sera. These multiplex PCR - REA protocols can provide accurate results as that obtained from sequence and phylogenetic analysis. Therefore, it can be used for fingerprinting the vaccine strains of PM, and subsequently will support the production of polyvalent vaccines and could be used instead of reference antisera when they are unavailable.

## Authors’ Contributions

AMA designed and supervised the project. DAMAE and NAE carried out the molecular biology section while EFE, ESAZ, and HAF performed the bacteriological isolation and biochemical tests. DAMAE wrote the manuscript with support from Dr. Abbas and AAS contributed to the final version of the manuscript. All authors read and approved the final manuscript.

## References

[1] Nascimento VP, Gama N.M.S.Q, Canal CW, Berchieri A, Silva EN, Di Fábio J, Sesti L, Zuanaze MAF (2009). Coriza infecciosa das galinhas, pasteureloses e outras infecções bacterianas relacionadas. Doenças das Aves.

[2] Khoo E, Khoo LL, Noormah MA, Zamila Z, Nafizah M, Sitinor HR, Saifu NR, Rosnah Y, Fhitri M, Roseliza R (2017). Capsular serogroup of *Pasteurella multocida* isolated in VRI, Malaysia from the year 2014 to 2016. Malaysian J. Vet. Res.

[3] Rigobelo EC, Blackall PJ, Maluta RP, Ávila FA (2013). Identification and antimicrobial susceptibility patterns of *Pasteurella multocida* isolated from chickens and Japanese quails in Brazil. Braz. J Microbiol.

[4] Carter GR (1990). Diagnosis of hemorrhagic septicemia. In: Veterinary Diagnostic Bacteriology. A Manual of Laboratory Procedures for Selected Diseases of Livestock.

[5] Heddleston KL, Gallagher JE, Rebers PA (1972). Fowl cholera: Gel diffusion precipitin test for serotyping *Pasteurella multocida* from avian species. Avian Dis.

[6] PHE (2015). UK standards for microbiology investigations identification of *Pasteurella* species and morphologically similar organisms. Standards unit, microbiology services. PHE Bacteriol.

[7] Ranjan R, Panda SK, Acharya AP, Singh AP, Gupta MK (2011). Molecular diagnosis of haemorrhagic septicaemia - A review. Vet. World.

[8] Jabbari AR, Esmaelzadeh M, Moazeni J.G.R (2006). Polymerase chain reaction of *Pasteurellamultocida* capsules isolated in Iran. Iran J. Vet. Res.

[9] Dziva F, Muhairwa AP, Bisgaard M, Christensen H (2008). Diagnostic and typing options for investigating diseases associated with *Pasteurella multocida*. Vet. Microbiol.

[10] Ghanizadeh A, Jabbari AR, Shayegh J, Sanchuli A, Banihashemi R (2015). Genotyping of *Pasteurella multocida* ovine and bovine isolates from Iran based on PCR-RFLP of *ompH* gene. Arch. Razi Inst.

[11] Ranjbar R, Karami A, Farshad S, Giammanco GM, Mammina C (2014). Typing methods used in the molecular epidemiology of microbial pathogens: A how-to guide. New Microbiol.

[12] Townseed KM, Frost AJ, Lee CW, Papadimitriou JM, Dawkins H.J.S (1998). Development of PCR assays for species-and type-specific identification of *Pasteurella multocida* isolates. J. Clin. Microbiol.

[13] Balakrishnan G, Roy P (2012). Isolation, identification and antibiogram of *Pasteurella multocida* isolation of avian origin. Tamilnadu J. Vet. Anim. Sci.

[14] Townseed KM, Boyce JD, Chung JY, Frost AJ, Adler B (2001). Genetic organization of *Pasteurellamultocida* cap loci and development of a multiplex capsular PCR typing system. J. Clin. Microbiol.

[15] Furian TQ, Borges K.A, Pilatti RM, Almeida C, Nascimento VP, do Salle C.T.P, Moraes H.L.S (2014). Identification of the capsule type of *Pasteurella multocida* isolates from cases of fowl cholera by multiplex PCR and comparison with phenotypic methods. Braz. J Poultry Sci.

[16] Mostafa AM, Seemann T, Gladman S, Adler B, Harper M, Boyce JD (2015). Comparative genomic analysis of Asian haemorrhagic septicaemia-associated strains of *Pasteurella multocida* identifies more than 90 haemorrhagic septicaemia-specific genes. PLoS One.

[17] Luo Y, Zeng Q, Glisson JR, Jackwood MW, Cheng I.H.N, Wang C (1999). Sequence analysis of *Pasteurella multocida* major outer membrane protein (*OmpH*) and application of synthetic peptides in vaccination of chickens against homologous strain challenge. Vaccine.

[18] Somshekhar SH, Veeregowda BM, Suryanarayana VVS, Leena G, Dhama K, Chakraborty S (2014). Outer membrane protein (OMP) profiles of *Pasteurella multocida* Isolates associated with haemorrhagic septicaemia by SDS-PAGE and Western Blot analysis. Asian J. Anim. Vet. Adv.

[19] Dogra V, Verma S, Singh G, Wani AH, Chahota R, Dhar P, Verma L, Sharma M (2015). Development of OMP based indirect ELISA to gauge the antibody titers in bovines against *Pasteurella multocida*. Iran. J. Vet. Res.

[20] Jabbari AR (2005). Molecular typing of avian *Pasteurella multocida* isolates by PCR-RFLP of *ompH* gene. Iran J. Biotechnol.

[21] Antony P, Nair G, Jayaprakasan V, Mini M, Aravindakshan T (2007). Nucleic acid based differentiation of *Pasteurella multocida* serotypes. J. Dairy Sci.

[22] Glisson JR, Saif YM (2008). Pasteurellosis and others respiratory bacterial infection. Diseases of Poultry.

[23] Geneidy AA, El-Affandy AM (1963). A Study of Past. Strains Isolated from Field Cases During the Last EIGHT years. Egypt 4th Arab. Anna Veterinary Cong., Cairo, Egypt.

[24] Carter GR, Bergan T (1984). Serotyping of *Pasteurella multocida*. Methods in Microbiology.

[25] Luo Y, Glisson JR, Jackwood MW, Hancock RE, Bains M, Cheng IN, Wang C (1997). Cloning and characterization of the major outer membrane protein gene (*ompH*) of *Pasteurella multocida* X-73. J. Bacteriol.

[26] Varte Z, Dutta TK, Roychoudhury P, Begum J, Chandra R (2014). Isolation, identification, characterization and antibiogram of *Pasteurella multocida* isolated from pigs in Mizoram with special reference to progressive atrophic rhinitis. Vet. World.

[27] Miflin JK, Blckall PJ (2001). Development of a 23S rRNA-based PCR assay for the identification of *Pasteurella multocida*. Lett. Appl. Microbiol.

[28] Davies RL, MacCorquodale R, Reilly S (2004). Characterization of bovine strains of *Pasteurella multocida* and comparison with isolates of avian, ovine and porcine origin. Vet. Microbiol.

[29] Davies RL (2004). Genetic diversity among *Pasteurella multocida* strains of avian, bovine, ovine and porcine origin from England and Wales by comparative sequence analysis of the 16S rRNA gene. Microbiology.

[30] Lee J, Kim YB, Kwon M (2007). Outer membrane protein H for protective immunity against. Pasteurella multocida.J. Microbiol.

[31] Priyadarshini A, Kumar S, Gupta SK, Viswas KN, Agarwal RK, Singh VP (2014). Cloning and sequence analysis of *hsf* an outer membrane protein gene of *Pasteurella multocida* serotype B:2. Vet. World.

[32] Okay S, Kızıldoğan AK (2015). Comparative genome analysis of five *Pasteurella multocida* strains to decipher the diversification in pathogenicity and host specialization. Gene.

[33] Nefedchenko AV, Glotova TI, Glotov AG, Ternovoy VA, Sementsova AO (2017). Prevalence of different OmpH-types among *Pasteurella multocida* isolated from lungs of calves with respiratory problems. Microb.Pathog.

[34] Singh R, Tewari K, Packiriswamy N, Marla S, Rao V.D.P (2011). Molecular characterization and computational analysis of the major outer membrane protein (*ompH*) gene of *Pasteurella multocida* P52. Vet. Arhiv.

[35] Johnson TJ, Abrahante JE, Hunter SS, Hauglund M, Tatum FM, Maheswaran S. K, Briggs RE (2013). Comparative genome analysis of an a virulent and two virulent strains of avian *Pasteurella multocida* reveals candidate genes involved in fitness and pathogenicity. BMC Microbiol.

